# An Antimicrobial Blue Light Prototype Device Controls Infected Wounds in a Preclinical Porcine Model

**DOI:** 10.1093/infdis/jiae548

**Published:** 2024-11-13

**Authors:** Laisa Bonafim Negri, William Farinelli, Sandeep Korupolu, Ying Wang, Yara Mannaa, Hang Lee, Jie Hui, Pu-Ting Dong, Andrea Slate, Joshua Tam, R Rox Anderson, Seok-Hyun Andy Yun, Jeffrey A Gelfand

**Affiliations:** Wellman Center for Photomedicine, Massachusetts General Hospital, Boston, Massachusetts, USA; Vaccine and Immunotherapy Center, Division of Infectious Diseases, Massachusetts General Hospital, Boston, Massachusetts, USA; Massachusetts General Hospital, Department of Dermatology, Boston, Massachusetts, USA; Harvard Medical School, Department of Dermatology, Boston, Massachusetts, USA; Wellman Center for Photomedicine, Massachusetts General Hospital, Boston, Massachusetts, USA; Massachusetts General Hospital, Department of Dermatology, Boston, Massachusetts, USA; Wellman Center for Photomedicine, Massachusetts General Hospital, Boston, Massachusetts, USA; Massachusetts General Hospital, Department of Dermatology, Boston, Massachusetts, USA; Wellman Center for Photomedicine, Massachusetts General Hospital, Boston, Massachusetts, USA; Massachusetts General Hospital, Department of Dermatology, Boston, Massachusetts, USA; Wellman Center for Photomedicine, Massachusetts General Hospital, Boston, Massachusetts, USA; Vaccine and Immunotherapy Center, Division of Infectious Diseases, Massachusetts General Hospital, Boston, Massachusetts, USA; Massachusetts General Hospital, Department of Dermatology, Boston, Massachusetts, USA; Massachusetts General Hospital, Department of Dermatology, Boston, Massachusetts, USA; Harvard Medical School, Department of Dermatology, Boston, Massachusetts, USA; Massachusetts General Hospital, Department of Medicine, Boston, Massachusetts, USA; Harvard Medical School, Department of Medicine, Boston, USA; Wellman Center for Photomedicine, Massachusetts General Hospital, Boston, Massachusetts, USA; Massachusetts General Hospital, Department of Dermatology, Boston, Massachusetts, USA; Harvard Medical School, Department of Dermatology, Boston, Massachusetts, USA; The ADA Forsyth Institute, Center for Comparative Medicine, Massachusetts General Hospital, Boston, Massachusetts, USA; Center for Comparative Medicine, Massachusetts Institute of Technology, Boston, USA; Wellman Center for Photomedicine, Massachusetts General Hospital, Boston, Massachusetts, USA; Massachusetts General Hospital, Department of Dermatology, Boston, Massachusetts, USA; Harvard Medical School, Department of Dermatology, Boston, Massachusetts, USA; Wellman Center for Photomedicine, Massachusetts General Hospital, Boston, Massachusetts, USA; Massachusetts General Hospital, Department of Dermatology, Boston, Massachusetts, USA; Harvard Medical School, Department of Dermatology, Boston, Massachusetts, USA; Wellman Center for Photomedicine, Massachusetts General Hospital, Boston, Massachusetts, USA; Massachusetts General Hospital, Department of Dermatology, Boston, Massachusetts, USA; Harvard Medical School, Department of Dermatology, Boston, Massachusetts, USA; Wellman Center for Photomedicine, Massachusetts General Hospital, Boston, Massachusetts, USA; Vaccine and Immunotherapy Center, Division of Infectious Diseases, Massachusetts General Hospital, Boston, Massachusetts, USA; Massachusetts General Hospital, Department of Dermatology, Boston, Massachusetts, USA; Harvard Medical School, Department of Dermatology, Boston, Massachusetts, USA

**Keywords:** antimicrobial blue light, antimicrobial resistance, *Staphylococcus aureus;* MRSA, phototherapy, wound infection

## Abstract

We developed a translational prototype antimicrobial blue light (ABL) device for treating skin wounds with ABL. Partial-thickness surgical wounds were created in live swine (an animal whose skin is considered the most like human skin), then heavily contaminated and left untreated for 24 hours with methicillin-resistant *Staphylococcus aureus* (MRSA). ABL treatment stabilized and reduced MRSA infection by greater than 4 orders of magnitude (>99.99%; *P* < .0001) compared with untreated wounds in the same animal, after only 2 daily treatments. These data support further development of such devices for controlling infection in skin wounds. ABL, with or without concomitant administration of negative pressure, antimicrobials, or photosensitizers, could play an important role in modern wound care by reducing the amount, duration, and cost of antibiotics needed, helping reduce antimicrobial resistance. No such device for treating human cutaneous wounds currently exists. This deserves further development and study.

## Teaser

A prototype antimicrobial blue light (ABL) device designed for clinical use was tested on porcine split-thickness wounds infected with methicillin-resistant *Staphylococcus aureus* (MRSA). ABL reduced MRSA by >99.99% after 4 treatments, potentially lowering antibiotic requirements for wounds.

We developed a prototype clinical antimicrobial blue light (ABL) device we hypothesize could add a useful new dimension to cutaneous wound care. Cutaneous wound infections are commonly associated with biofilms that both protect bacteria from antibiotics and slow wound healing [1, 2]. Biofilm infections are notoriously more resistant to antibiotics (100- to 1000-fold) than sensitivity tests predict, acting as a protective carapace, enabling metabolic changes favoring persistence [3] of bacteria, and due to their chronicity are major sources generating multidrug resistant (MDR) bacteria.

ABL (400–470 nm) has emerged as a potential strategy complementary to antibiotics against MDR cutaneous infection. ABL, specifically at 405 nm, is effective against a wide variety of pathogenic microorganisms, in planktonic bacteria and biofilm culture. The antimicrobial mechanism for this effect involves ABL absorption by endogenous chromophores in bacterial cells, which promote photochemical reactions resulting in production of reactive oxygen species (ROS) intracellularly, leading to bacterial cell death. Despite knowledge of antimicrobial activity of ABL in vitro and in vivo in small animal models, no controlled investigation has been performed applying ABL in significant cutaneous wound infections in large animals [4–6], necessary before human study. Additionally, antibiotic resistance does not confer resistance to the bactericidal effects of ABL.

We believe an ABL device will not replace antibiotic therapy, rather augment it, reducing total doses of antibiotics and time required to treat a wound, especially biofilm wounds. Reducing antibiotic dosing and duration should reduce the potential for development of antimicrobial resistance (AMR), duration and cost of treatment, and drug toxicities. Successful integration of ABL could be useful in antibiotic stewardship efforts to limit antibiotic use [7].

Our device is based on decades of work by others [4, 6, 8–11], as well as recent work of our own, investigating the effects of ABL on bacteria in vivo in small animal infection models, as well as ex vivo porcine wound model skin experiments [3]. There have been human studies using very-low-power blue light to treat acne, as well as gingival or root canal infections treated with high-power ABL, but currently there are no US Food and Drug Administration (FDA) or European Union registered devices for significant cutaneous wound infections [5].

In this report, we describe a porcine, preclinical model of partial thickness, established, high-bioburden methicillin-resistant *Staphylococcus aureus* (MRSA) wound infection, which on histopathologic analyses are biofilm infections. The porcine model is the animal model of choice for testing treatments for wound infections before human studies [12–14]. In our wound model, our device reduced MRSA bacterial burden significantly after only a single daily ABL treatment when compared with untreated wounds. MRSA was chosen because it is the leading AMR wound pathogen isolated worldwide, and also one of the common wound pathogens *least sensitive* to ABL [3]. Thus, effectiveness against MRSA should be predictive of utility against a broader spectrum of other bacterial wound pathogens, an assumption well supported by previous studies [5, 8].

## METHODS

### Device

**Figure 1.**
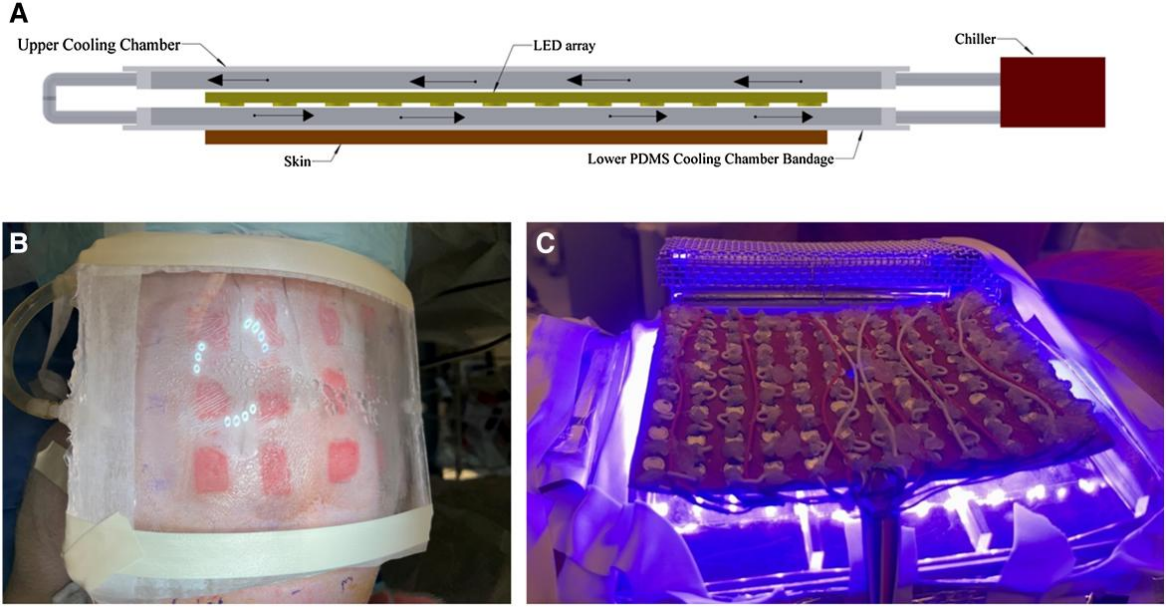
*A*, Schematic representation of the prototype device that was used in the porcine experiments. In this second prototype, we have added the heat exchange circuit. This includes a flexible and translucent PDMS chamber above the wound. *B*, Recirculating cooling chamber. *C*, PDMS bandage with cooling chamber on the porcine wounds. Abbreviations: LED, light-emitting diode; PDMS, polydimethylsiloxane.

The dressing was polydimethylsiloxane (PDMS), an already FDA-approved material for contact with human tissue used in disposable contact lenses, made in the dimensions of an abdominal (ABD) pad, 13 x 18 cm. The prototype comprised 3 major components: an illuminating component with 405-nm light-emitting diodes (LEDs), power circuitry, and an electronic controller device; a dressing component in direct contact with the wound, transparent and resistant to fouling; and a cooling circuit to dissipate heat generated by the LEDs. A schematic for the design is in Figure 1*A*. The PDMS cooling chamber and the heat sink of LEDs are shown in Figure 1*B.* The lighted PDMS bandage without the top cooling chamber is shown in Figure 1*C*. We placed 3 thermocouples at different locations under the bandage, adjacent to the wounds, and continuously monitored the surface temperature of the skin throughout the treatment. The cooling circuit integrated into our bandage enabled us to maintain a stable skin surface temperature at approximately 35°C ± 1°C to minimize potential thermal effects and ensure safety. The heat sink of the LEDs was maintained between 65°C and 70°C. The entire device was flexible and conformed to the curved areas of treatment zones, ensuring uniform cooling and illumination. A detailed description of the device design is in Supplementary Material, Supplementary Figures 1 and 2.

### Bacterial Strain and Growth Conditions

The strain used in this study was MRSA USA300 [15], as in antecedent porcine ex vivo MRSA-infected wound model experiments used for predicting ABL dosimetry [3]. This was cultured on brain heart infusion (BHI) agar plates at 37°C in 5% CO_2._ After obtaining colonies from the BHI agar plates, bacterial suspensions were grown in a shaking incubator overnight in BHI broth, then collected by centrifugation at 4000 rpm for 10 minutes and resuspended at a density of 1 × 10^8^  colony-forming units (CFU)/mL. After the wound biopsies, all inocula were similarly grown and quantitated after growth on Becton Dickinson BBL CHROMagar MRSA II selective media. We used this check to ensure that we were inoculating, recovering, and quantitating MRSA.

### The Porcine Wound Model

We adopted features from several recent porcine infection models [16–19]. Our protocol (No. 2022N00048) was approved by the Massachusetts General Hospital Institutional Animal Care and Use Committee and the Animal Care and Use Review Office. We used 3 female Yorkshire swine (38–45 kg) over the course of the current study.

Prior to surgery, animals were fasted overnight, then sedated, intubated, and isoflurane anesthesia administered. Hair was clipped and animals were prepped and draped using standard surgical techniques with final cleansing by iodine and 70% ethanol.

Partial-thickness wounds were created with a surgical dermatome, 2.5 cm wide by 1 cm long, 300 µm deep, with wounds at least 2.5 cm separated from adjacent wounds. This created wounds into the dermis, with epidermis entirely removed. Hemostasis was established with sterile cotton dressings covered by sterile Tegaderm. Wounds were placed on both sides of the dorsal chest of each pig, separated by 2 different zones: a blue light zone and a dark zone (control), as seen in Supplementary Figures 3 and 4, and Supplementary Tables 1 and 2; details of wound sampling technique are there as well.

After wounds were placed and hemostasis achieved, all wounds were infected by inoculating 25 µL of MRSA at a concentration of 1 × 10^8^ CFU/mL, resulting in an inoculum 2.5 × 10^6^ CFU per individual wound. A sterile inoculation loop was used to gently spread the inoculum across entirety of the 2.5 cm × 1 cm wound area. After no more fluid was seen in the wound, it was covered with sterile Tegaderm individually. All wounds were created and inoculated on day 1 and wound infection groups with different time points were established (24, 48, 72, and 96 hours postinfection). Due to MRSA infection of the wounds, our Institutional Animal Care and Use Committee required euthanasia at the end of the 96-hour procedures. At all times during wounding and infection experiments, animals were under either general anesthesia or narcotized; upon awakening from anesthesia and having received narcotics, they behaved normally.

The MRSA biofilms in the wounds on the light side were treated with ABL only once daily (40 mW/cm^2^; 250 J/cm2; 104 minutes) in all groups; ABL treatment started in all wounds 24 hours postinfection. Sterile thermocouples were placed immediately adjacent to the wounds. After treatments, 3 punch biopsies (4 mm) were harvested to quantify CFU/g of MRSA for each group, in both dark and ABL zones.

### Histological Evaluation

The full-thickness skin of 4 mm punch biopsies harvested for each group were fixed in 10% formalin. Samples were cut into half and paraffin-embedded (5 μm thickness) in cell culture glass slides. The samples were stained with a combination of hematoxylin and eosin (H&E) and by modified Brown-Hopps tissue Gram stain technique [20]. Samples were scanned using a NanoZoomer slide scanner (Hamamatsu) for evaluation.

### Fluorescence In Situ Hybridization Assay

A protocol from prior studies [21] was followed, using an Alexa488-tagged peptide nucleic acid fluorescence in situ hybridization (FISH) probe to specifically target *S. aureus.*

### Statistical Methods

The responses from dark (control) and ABL wounds were obtained in the same animal, in replicates, at the same time point, which would minimize the between-animal variation by using animals as ethically and economically as possible. Mixed effects model ANOVA for repeated measures (MM-ANOVA-RM) was applied to compare the mean levels of log_10_ CFU/g of MRSA across the multiple postinfection time points. The model's random effects were the animal level intercepts and the fixed effects were those of the postinfection time lengths. The specified longitudinal error structure of the model was compound symmetry. The longitudinal means and their standard errors as well as the individual data points were displayed and were graphically described with the statistical significance obtained from the MM-ANOVA-RM.

## RESULTS

The results of using the initial device resulted in thermal damage to the skin (Supplementary Figure 5), and the anesthetized animal was euthanized. We then reengineered the device to include cooling circuits. The addition of the cooling circuits maintained the skin immediately adjacent to the wound always between 34°C and 35°C.

Based on our prior evaluation of ABL dosimetry in vitro and in an ex vivo porcine skin wound model, we had determined that in both of these models, 250 J/cm² (40 mW/cm^2^) was a generally effective dose/fluence of ABL to kill most wound pathogens previously evaluated in our biofilm and wound model [3]. In the present study, this dose was used for wound treatment in the animals. The untreated dark side was draped during illumination treatment with a large sterile Tegaderm dressing that was, in turn, covered with aluminum foil to prevent adjacent ABL light from reaching these wounds.

As previously noted, every wound infected received an inoculum of 2.5 × 10^6^ CFU of MRSA at time 0 and the growth curve in the untreated infected wounds was determined every 24 hours (Supplementary Figure 6). In our previously published ex vivo porcine skin wound model, this produced biofilm infections [3]. It is against this untreated growth of the huge MRSA bioburden in the same animal that the ABL device treatments were then compared at each time point postinfection in the same animal.

**Figure 2.**
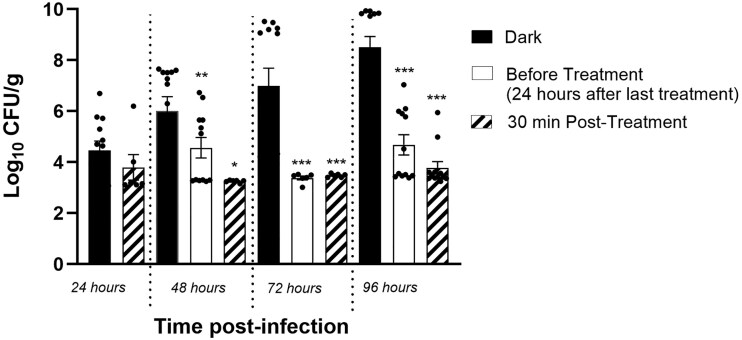
The longitudinal logarithmic means and their standard errors as well as the individual data points are displayed graphically for each group of wounds postinfection (24, 48, 72, and 96 hours). The black bars show the log CFU/g of MRSA in the dark control (untreated zone); white bars the log CFU/g of MRSA in the wounds before the ABL daily treatment (ie, 24 hours after the previous treatment); and white with pattern bars the log CFU/g of MRSA in the wounds after ABL daily treatment (30 minutes posttreatment). The statistical comparisons between untreated and treated wounds were performed using with mixed effects model ANOVA for repeated measures. *P < .01, **P < .001, ***P < .0001. The distributions of 30-minute posttreatment log_10_ CFU/g at 72 and 96 hours postinfection are completely separated (ie, no overlaps) from those of dark, untreated control conditions. Abbreviations: ABL, antimicrobial blue light; CFU, colony-forming units; MRSA, methicillin-resistant *Staphylococcus aureus.*

The first ABL treatment started 24 hours postinfection in all wounds in the blue light zone and was applied once a day. In pig No. 2, biopsies were collected 24 hours after each ABL treatment to analyze the effect of ABL on infected wounds the day after treatment. In pig No. 3, the biopsies were additionally obtained 30 minutes after ABL treatment to analyze the effect of ABL shortly after exposure. With this modification we were able to compare the CFU of MRSA before the daily treatment (ie, 24 hours after the previous treatment; Figure 2, orange bars), and after the daily treatment (30 minutes posttreatment; blue bars) for each group of wounds postinfection, in addition to comparing these with dark controls (Figure 2, gray bars). In all postinfection groups, ABL-treated wounds demonstrated highly significant log CFU/g reductions in MRSA compared to dark controls, at both times postirradiation (24 hours and 30 minutes). Treated samples collected 24 hours postirradiation showed a reduction of 1.5 log_10_ (Figure 2). After the fourth treatment, at 96 hours postinfection, a 4.7 log_10_ CFU/g reduction was seen. By 72 and 96 hours, the reductions were highly significant at *P* < .0001 (Figure 2 and Supplementary Table 3).

In addition, we made the conscious decision not to debride purulence from any of the wounds, as that would create a subjective artifact. Lack of debridement of the wound, allowing exudate to remain, decidedly working against the antibacterial activity of ABL by quenching photon energy reaching the wound, and thus diminishing its efficacy. The absence of active wound debridement likely contributed to the fact that at every time point, there was a residual bacterial bioburden that was consistently untouched for 24 hours, which may have been deeper in the tissue and beyond the reach of blue light but contained in the punch biopsy core that was cultured, which extended to almost 4 mm.

### Histopathology

**Figure 3.**
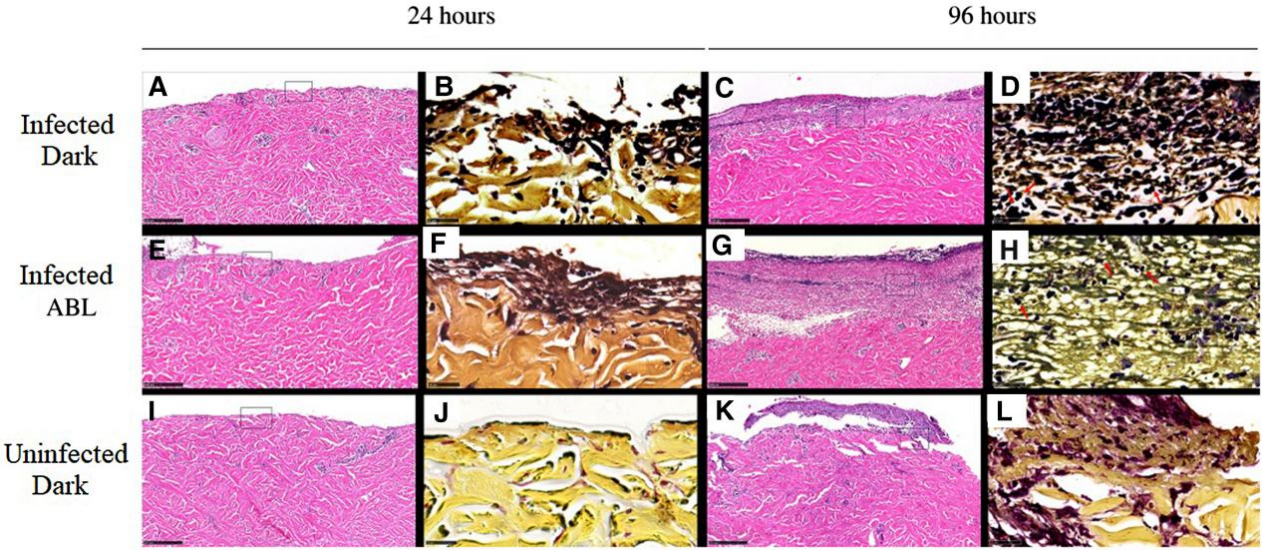
The left 2 columns (*A*, *B*, *E*, *F*, *I*, and *J*) are biopsy specimens obtained 24 hours after wounding; the right 2 columns (*C*, *D*, *G*, *H*, *K*, and *L*) are biopsy specimens obtained 96 hours after wounding, from within the partial thickness wound bed, lacking epidermis. Specimens were H&E stained (*A*, *E*, *I*, *C*, *G*, and *K*) or Gram stained (Brown and Hopps) (*B*, *F*, *J*, *D*, *H*, and *L*). The rows show biopsies of wounds after various treatments: top, wounded and infected, untreated (*A*, *B*, *C*, and *D*); middle, wounded, infected, and ABL-treated (*E*, *F*, *G*, and *H*); and bottom, wounded, uninfected, and untreated (*I*, *J*, *K*, and *L*). Comparing (*B*) infected, untreated to (*F*) infected, ABL-treated, 24 hours postinfection, the number of gram-positive cocci appears approximately the same with and without treatment. By 96 hours, there was substantial gram-positive cocci infiltration in the 96-hour untreated sample, including diffuse gram-positive staining of indistinct material compatible with, but not diagnostic of, peptidoglycan of an MRSA biofilm (*D*). There was some, but not nearly as much, gram-positive cocci infiltration in the ABL-treated sample (*H*). Crystal violet of the Gram stain is taken up by the proliferating, reepithelializing tissue in the wounded, uninfected biopsy (*L*), presumably from uptake of dye by nuclear material of the dermal cells in the granulation tissue. *D* and *H*, Red arrows indicate classical *Staphylococcus aureus* gram-positive cocci as single, clumps, and short chains. H&E scale bar 250 µm; Gram stain scale bar 25 µm. Abbreviations: ABL, antimicrobial blue light; H&E, hematoxylin and eosin; MRSA, methicillin-resistant *Staphylococcus aureus.*

The infected wounds, both with ABL treatment and without ABL treatment, had more gram-positive staining than the control uninfected wounds initially at 24 hours and finally at 96 hours postinfection (Figure 3*B*, 3*F*, 3*J*, 3*D*, 3*H*, and 3*L*). By visual inspection of the concomitant H&E sections (Figure 3*A*, 3*E*, 3*I*, 3*C*, 3*G*, and 3*K*), the ABL-treated infection group has substantially less infiltration of neutrophils than the untreated, infected group, consistent with less infection. The wounded, control group with no infection/no ABL began to reepithelialize at 72 hours postwounding.

**Figure 4.**
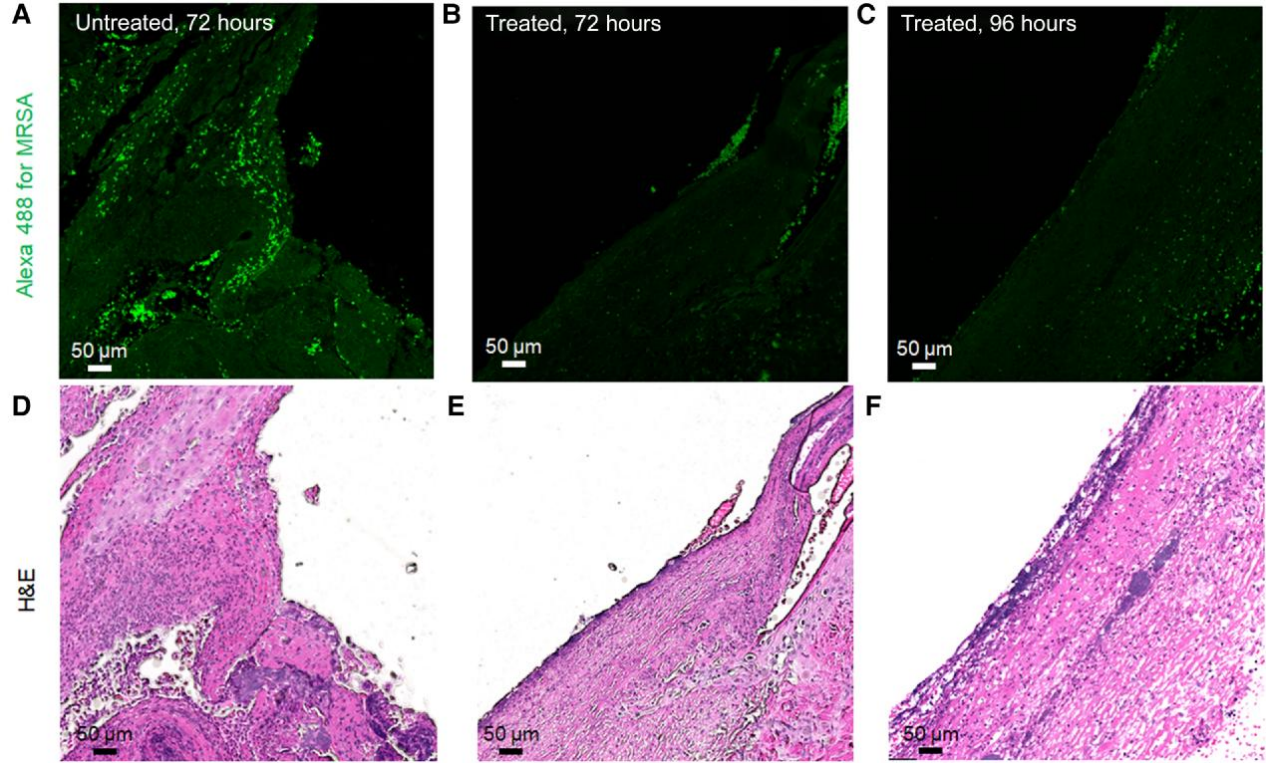
Visualization of bacterial presence in the in vivo wound infection by *Staphylococcus aureus*-specific PNA-FISH imaging assay (*A*, *B*, and *C*) and corresponding H&E-stained histological sections (*D*, *E*, and *F*) from each group. Green fluorescence indicates the presence of bacteria. *A* and *D*, 72 hours biofilm untreated (dark); *B* and *E,* 72 hours biofilm with 3 ABL treatments, once per day; *C* and *F*, 96 hours biofilm with 4 ABL treatments, once per day. The thick fluorescent band in the untreated 72-hour wound infection (*A*) is highly suggestive of a biofilm. The progressive loss of fluorescence intensity by PNA-FISH is a qualitative demonstration of the efficacy of the treatments. Abbreviations: ABL, antimicrobial blue light; H&E, hematoxylin and eosin; PNA-FISH, peptide nucleic acid-fluorescence in situ hybridization.

The CFU bioburden in control and treated wounds was visualized by FISH assay using *S. aureus*-specific peptide nucleic acid probes. The intensity of fluorescence observed in untreated infected wounds at 72 hours is strongly suggestive of MRSA biofilm (Figure 4*A*), which substantially decreased visually when the wounds were treated with ABL (Figure 4*B* and 4*C*).

## DISCUSSION

In this preclinical, translational experiment, we have demonstrated that ABL exposure significantly and greatly limits the burden of a drug-resistant pathogen (MRSA) in contaminated wounds, in a large animal model with skin very similar to humans. A prototype system for blue-light treatment was designed and used, which meets multiple criteria for being practical, safe, and affordable.

The antibacterial effect of light upon bacteria was first documented in controlled experiments by Downes and Blunt, and published in 1877 [22]. Photodynamic therapy of bacteria was described by Von Tappeiner in 1907, from experiments with microbes, light, and eosin, showing that oxygen was necessary [23]. By 1986, it was reported that blue light (409 nm) was capable of interacting with porphyrins within *Propionibacterium acnes* bacteria [24], and similar observations were made with *S. aureus* [25]. In the past 80 years, development of new antibiotics has slowed to a trickle, while AMR has become pervasive.

Over the past 20 plus years, investigators have explored the effects of the blue light spectrum (400–470 nm) on the eradication of a variety of pathogenic bacteria [5, 6, 26]. These investigators have established that the ABL is microbicidal due to absorption of photons by endogenous bacterial chromophores acting as endogenous photosensitizers. Absorption of light by these chromophores develops an excited molecular state, transferring electrons to oxygen molecules that generate ROS within the bacteria themselves [5, 8]. Light-generated ROS then interferes with bacterial DNA, lipids, efflux pumps, cell membranes, and walls, resulting in death of the bacteria. There is a correlation between ABL sensitivity in bacteria and the presence of endogenous microbial chromophores, including porphyrins, flavins, and other chromophores, particularly coproporphyrin [8], but no correlation with antibiotic resistance. The nature and quantity of the endogenous chromophores, as well as the bacterial antioxidant defenses, determine the sensitivity of various species of microbes to ABL [5]. Mammalian cells appear to be more resistant to the ROS-generating effects of blue light [26, 27]. Other advantages of ABL include the virtual absence of development of resistance to light by bacteria and the wide, though variable sensitivity to ABL of virtually all wound pathogens, enabling potential empirical use of ABL without testing for sensitivity [5, 11, 26].

Despite the advantages of treatment of cutaneous wounds with ABL, no devices employing ABL have been approved for use for the treatment of cutaneous wounds in the clinic [26]. ABL treatments have found their way into clinical use for gingivitis and acne [5, 8, 27, 28], but to date no approved phototherapy devices have emerged for infected skin wounds. In fact, no successful study of ABL treatment of infected skin wounds in a large-animal, swine model has yet been published to our knowledge, the necessary final step before human clinical trials and regulatory approval. Our current research has revealed 2 likely reasons why: the significant light doses required for effective treatment of sizable, infected wounds in large animals result in both heat generation and conduction of the heat by the LEDs in the conversion of the electrical energy to light. The second reason is poor tissue penetration of blue light.

Our experience with the thermal problem in our first swine model led us to a simple technology solution that makes ABL treatment a possible modality for addressing MDR biofilm infections in humans. For our experiments with the second and third animals, a cooling circuit was introduced in addition to the already present thermocouples. This enabled us to both monitor and control temperature, which we maintained at the skin level at approximately 35°C ± 1°C. With temperature in the wound regulated, we were able to deliver our intended fluence (dose) of ABL. This simple workaround should enable early adoption of this potentially useful adjunct therapy for wounds, as other mitigating strategies for improving thermal management are explored (duty cycle; addition of topical adjuvants as vitamin K_3_ and curcumin [3]). The second problem, poor tissue penetration by light, is a much more complicated problem that is largely unaddressed in these initial experiments.

Despite poor tissue penetration, the results for animals No. 2 and No. 3 were dramatic. Forty-eight hours after wound inoculation of over 2 million CFU MRSA into each wound, the MRSA burden in the untreated wounds was approximately 10^7^ CFU/g tissue. The MRSA bioburden grew to almost 10^9^ CFU/g tissue in the untreated wounds by 96 hours after inoculation. For both animals No. 2 and No. 3, after the second treatment, the geometric mean CFU/g of tissue was reduced by almost 4 logs compared to untreated wounds. At 96 hours the MRSA bacterial bioburden was down almost 5 logs compared to untreated wounds, despite our intentional lack of debridement, which would have increased quenching of ABL by purulence in the wound. ABL treatment alone demonstrated dramatic substantial therapeutic effects. A high level of statistical significance was demonstrated in only 2 animals by using each animal as its own experimental and control subject; this was achieved by comparing treated to untreated wounds in the same animal at the same time points, and so each animal served as its own control.

It is important to note that blue light is attenuated by optical absorption and scattering in dermis, with a half-value depth of approximately 400 µm [29]. As the epidermis had been removed by the microtome, our punch biopsy sampled to a depth of approximately 4 mm for the sampled tissue to be homogenized for quantitative culture (CFU/g), and included depths of dermis in which bacteria were readily seen histologically by Gram stain. The depth of 4 mm is approximately 10 half-value layers, and the 96-hour posttherapy colony count was about 0.1% of the untreated wound bacterial burden. With the surface fluence of 250 J/cm^2^, there would be about 250 mJ/cm^2^ at the 4 mm depth. This is not a lot of blue light, but not zero. Blue light potency is far less germicidal than wavelengths like 254 nm, for which 1 mJ/cm^2^ is highly lethal, but blue light is much more penetrating.

The profound reduction in bacterial load is likely due to a combination of the powerful photo-oxidative damage of the ABL at the surface of the wound where infection began, possibly involving a lesser contribution from the increase in activated neutrophils (polymorphonuclear neutrophils) deep to the site of the original infection, mediated by the neutrophil effects in the tissue [30]. The sum of these antimicrobial effects may contribute to the bacterial killing in the wound. The addition of potential ABL adjuvants to the wound such as vitamin K_3_ could add 1–3 additional logs of killing, at minimal cost, as our group has demonstrated in the porcine ex vivo wound model [3]. Furthermore, our experimental protocol, allowing 24 hours to elapse before the next treatment, strongly favored bacterial rebound growth. Several treatments per day would likely work even better. Additionally, we intentionally refrained from debriding the wounds, lest we introduce subjectivity into the natural evolution of these wounds.

We hypothesize that the light is having a greater killing effect on bacteria on the surface of the wound, especially bacteria in the biofilm that are reached by blue light, while systemic antibiotics would be acting from the vascularized tissue below. It is not hard to imagine that the combined therapeutic effects from above and below an infected biofilm wound could lead to reduced treatment time as well as reduced necessity for repeated antibiotic treatment if the 2 therapeutic modalities were to be combined simultaneously.

In conclusion, we have demonstrated in proof-of-concept experiments using a preclinical translational porcine model of heavily contaminated MRSA wound infection, that ABL can produce multiple log reductions of bacterial burden using a relatively simple, safe, and inexpensive device. Our preliminary experiments also suggest that antimicrobial blue light may be effective in the tissue beyond the first half-value depth of tissue penetration of its photons. This hypothesis remains to be proven. Nonetheless, these observed effects, if further combined with systemic or topical antibiotics or other adjuvant chemicals, or as a stand-alone therapy, may prove a potent addition to how we treat skin and soft tissue infections, contributing to a reduction of antimicrobial resistance by decreasing total antibiotic dosage administered, representing a potentially new tool for antibiotic stewardship.

## Supplementary Data

Supplementary materials are available at *The Journal of Infectious Diseases* online (http://jid.oxfordjournals.org/). Supplementary materials consist of data provided by the author that are published to benefit the reader. The posted materials are not copyedited. The contents of all supplementary data are the sole responsibility of the authors. Questions or messages regarding errors should be addressed to the author.

## Supplementary Material

jiae548_Supplementary_Data

## Notes

***Acknowledgment.*** The authors gratefully acknowledge the staff of the Knight Animal Surgery Laboratory for their assistance.

***Data availability.*** Further data that support the findings of the study are available on request to the corresponding author.

***Financial support.*** This work was supported by Uniformed Services University (USU) and The Geneva Foundation (grant number HU00011920056 to Musculoskeletal Injury Rehabilitation Research for Operational Readiness, USU and The Wellman Center collaboration); National Center for Advancing Translational Sciences, NIH (grant number UM1TR004408 awarded through Harvard Catalyst, the Harvard Clinical, and Translational Science Center); and Harvard University and its affiliated academic healthcare centers. Additional support for J. A. G. was from the Edmund C. Lynch, Jr Research Fund. R. R. A was supported in part as the Lancer Endowed Chair in Dermatology at Massachusetts General Hospital.
