# Supplementary material for: An Antimicrobial Blue Light Prototype Device Controls Infected Wounds in a Preclinical Porcine Model
Source: J Infect Dis. 2024 Nov 13;231(3):e545–52. doi: 10.1093/infdis/jiae548 (PMC11911787; doi:10.1093/infdis/jiae548)
Supplement: jiae548_Supplementary_Data [file jiae548_supplementary_data.docx]

**SUPPLEMENTARY INFORMATION**

**Title:** An antimicrobial blue light prototype device controls infected wounds in a preclinical porcine model.

**Authors:** Laisa Bonafim Negri ^a, b, c, d^, William Farinelli ^a, c^, Sandeep Korupolu^a, c^, Ying Wang^a, c^, Yara Mannaa^a, b, c^, Hang Lee^c, d^, Jie Hui^a,c,d^, Pu-Ting Dong^e^, Andrea Slate^f^, Joshua Tam ^a,c,d^, R. Rox Anderson^a,c,d^, Seok-Hyun Andy Yun^a,c,d^, Jeffrey A. Gelfand^a,b,c,d,#^

^a^Wellman Center for Photomedicine, Massachusetts General Hospital (MGH), Boston, MA, USA;

^b^Vaccine & Immunotherapy Center, Division of Infectious Diseases, MGH, Boston, MA, USA;

^c^Massachusetts General Hospital, Boston, MA, USA;

^d^Harvard Medical School, Boston, MA, USA;

^e^The ADA Forsyth Institute; Center for Comparative Medicine, Massachusetts General Hospital, Boston, MA, USA.

**Short Title**: Blue light treatment of cutaneous wounds in swine

**Device Design:** For the illuminating component, we selected a substrate crafted from a silicone rubber sheet (McMaster Carr #1460N24, 1/8 inch thick) due to its exceptional flexibility, heat resistance up to 450^o^F, and autoclavable nature, rendering it reusable. Ninety-six 405 nm LEDs were then embedded into this substrate, inexpensively sourced (Chanzon Tech. Co., China, #AA0336, 3 Watt LEDs). These LEDs feature a domed front surface with a full-angle beam divergence of 130 degrees. The anode and cathode connections are positioned on opposite sides of the LED and a heat sink plate is located on the back. Considering the 6mm body diameter of the LEDs and their divergence angle, we determined that 96 LEDs spaced 1.5cm apart center to center, organized into 8 rows by 12 columns (Figure S1), are necessary to achieve a nominally uniform light distribution across the bandage. The LEDs were securely placed using flowable silicone adhesive, and the electrical connections were systematically wired in series and parallel. On the back side of the LEDs, we embedded 5x6mm lengths of copper rod using a two-part thermal conductive adhesive (MG Chemical #8328TCM-6ml). Surrounding each rod, we applied two layers of thermal heat sink padding (AIYUNNI silicone heat pad, 3mm thick) to facilitate uniform heat dissipation. The LEDs were run by a power supply in a constant current mode.

**
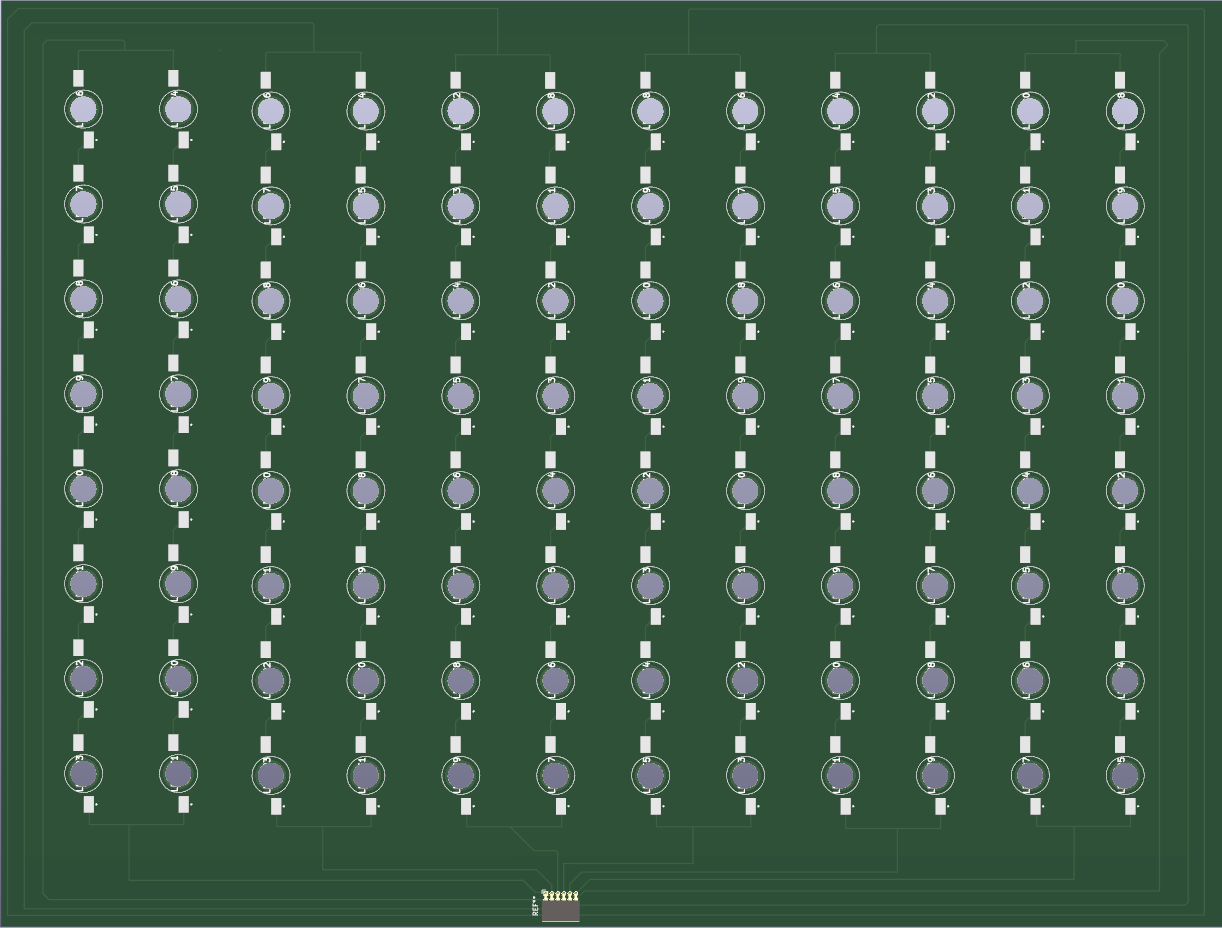
**

**Figure S1.** Schematic of placement of LEDs

The dressing component, which is in direct contact with the wound and requires efficient light transmission from the LEDs, demanded a material with specific attributes, as outlined in the TPP attributes listed above. After evaluating numerous materials, we selected polydimethylsiloxane (PDMS). PDMS, commonly used in the production of soft, disposable contact lenses, possesses transparency, elasticity, and the ability to readily conform to irregular shapes and surfaces. Additionally, it allows for easy modifications for future iterations. The bandage was made by casting PDMS into a 3-D printed mold (Figure S2) with the material RGD 450 on Stratasys poly-jet printer.


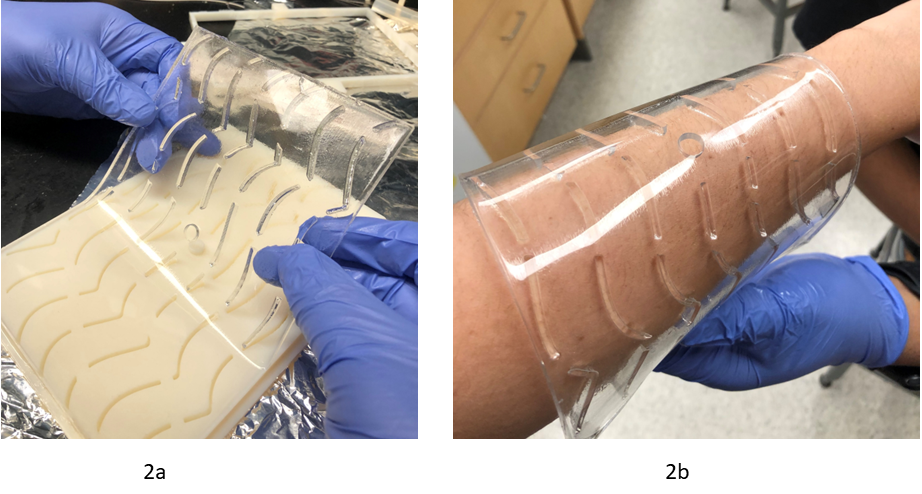


**Figure S2.** A. Removing casted PDMS bandage from mold. B-representation of transparency of the PDMS bandage.

In our experience with our first porcine subject (pig#1), we identified heat generation as a significant concern. Consequently, we implemented a straightforward yet effective cooling circuit system using temperature-controlled flowing sterile water. Two PDMS rectangular chambers, each measuring 5x7x0.75 inches (W, L, H), were created by casting PDMS into a 3D printed mold. On each end of the chamber, a ¼ inch diameter tubing connector was fitted. Two precision thermoelectric recirculating chillers (ThermoCube™) were utilized to circulate temperature-controlled water to the translucent, flexible chambers. This resulted in the formation of two cooling systems “sandwiching” the LED-holding layer—one at 30^o^C was placed in direct contact with the

tissue on one side and the illuminating component on the other side, and the second cooling chamber was placed on the back side of the illuminating component's LEDs at 2.5^o^C, further cooling the LEDs.

As our recirculating chiller had only one pump to deliver water to the chamber, water entered the chamber faster than it could be returned to the chiller, causing a "ballooning" effect. To address this, a 12-volts water pump was added to the return side of the chambers, and the incoming and outgoing flow rates were matched to ensure the chambers remained uniformly flat with good contact to the cooling targets.

To gain insight into the cooling system's functionality, we assessed heat absorption on a metal surface with our ABL device. We applied the bandage to an aluminum plate and monitored the temperature over 15 minutes. With the cooling activated, the temperature stabilized at approximately 18°C ± 1°C. In contrast, without cooling, the plate's temperature steadily rose from 18°C to 36°C. This experiment reinforces the cooling system's effectiveness in managing heat and preventing unwanted thermal buildup.

**Animal acclimation**: The pigs were quarantined for 7 days to acclimate. The animals were visited daily by the investigators and play enrichment and acclimation were provided through treats such as apples and yogurt as well as toys. On the day of surgery, the animals had been fasted overnight. Animals were initially treated with atropine (0.04 mg/kg IM, Telazol 4.4 mg/kg, xylazine 2.2 mg/kg IM), and then intubated at which point isoflurane anesthesia was begun. A 22-gauge left auricular vein IV catheter was placed. Then, the animals underwent close clipping of the hair over the dorsum of the spine in the thoracic area on both sides of the animal.

**Wounds design:**

Each group of post-infection wounds (ie., 24 hours, 48 hours, 72 hours, 96 hours) was biopsied in quadruplicate with 4mm punch biopsies of each individual 2.5x1cm wound only once, 3 punches for quantitative bacteriology and 1 for histology. In the ABL “time zones” on pig #2, we biopsied all wounds 24 hours post-irradiation. In the ABL “time zones” on pig #3, we biopsied 2 wounds 30 minutes post-irradiation and 2 wounds 24 hours post-irradiation. Each tissue sample was weighed and then placed in tissue grinder tubes. Tissue was homogenized for 30 min in a tissue homogenizer. An aliquot of 100 μl was removed and plated using a 10-fold dilution series to quantify CFU/g of tissue^3^. All the samples were inoculated and quantitated after growth on Becton Dickinson BBL CHROMagar™ MRSA II. The dark control (no ABL) wounds were analyzed in the same conditions and same time points of post-wound infection. Before light treatment or biopsy, the wounds were not debrided to remove exudate. After punch biopsies were obtained, wounds were covered with a dry-sterile dressing (DSD) and not touched again. Thus, for every time point post-infection that was sampled in a single animal, treated and untreated wounds were compared in that same animal at the same time point, each with 6-9 quantitative cultures/ time point by 4mm punch biopsy.


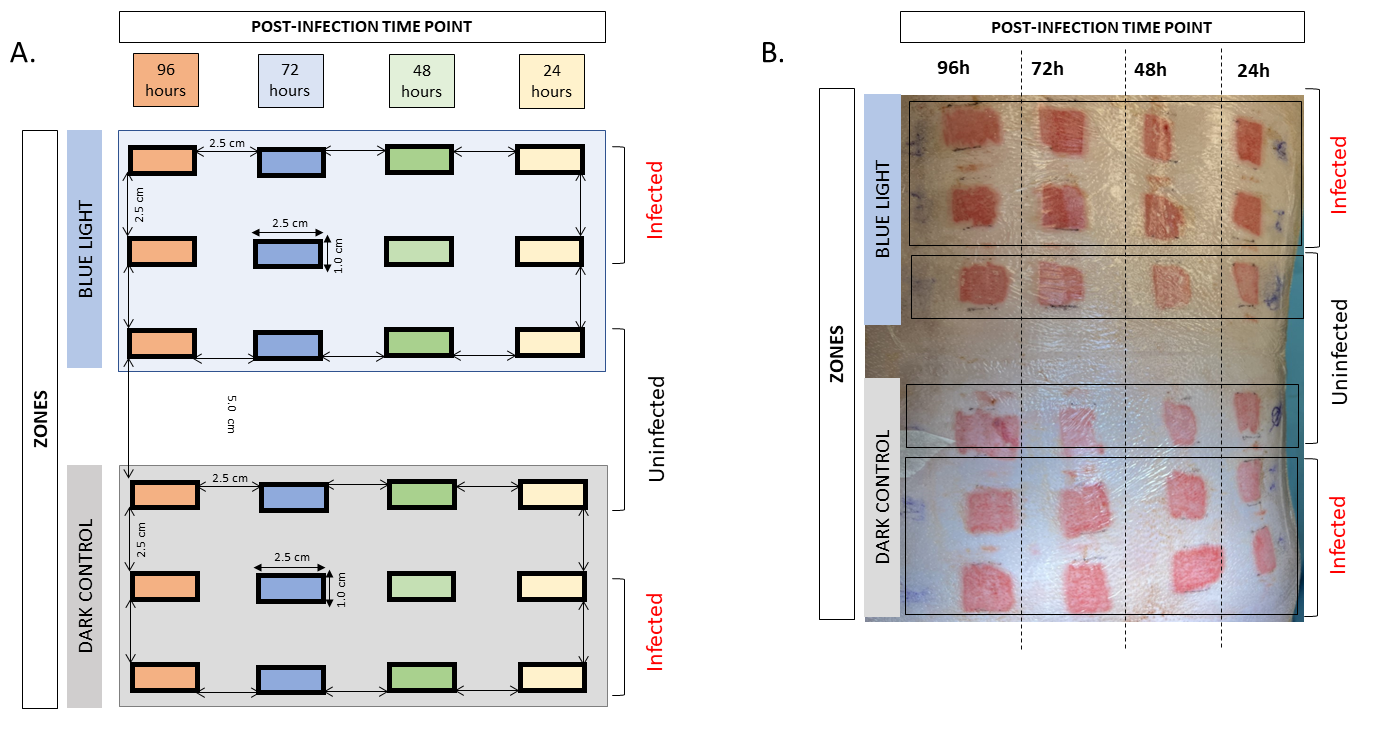


**Figure S3**. A total of 24 wounds were placed in pigs #1 and #2, separated by two different “zones”: blue light zone and dark zone (control). The flowchart (A) and the dorsal chest of the pig (B) show that the wounds were divided into four different groups to grow the bacteria biofilms at different time points post-infection (24h, 48h, 72, and 96h), each group contained 3 wounds for light irradiation (2 wounds infected and 1 wound non-infected) and 3 wounds for dark control (2 wounds infected and 1 wound non-infected).

**Table S1**. The table shows the number of wounds in pigs #1 and #2, in different zones (blue light zone and dark control zone) for each post-infection group (24h, 48h, 72h, 96h post-infection), over a total of 24 wounds.

| ***Groups***  ***( Time post-infection)*** | ***Zones of treatment*** | | | |
| --- | --- | --- | --- | --- |
|  | **Blue light zone** | | **Dark control zone** | |
|  | Infected | Non-Infected | Infected | Non-Infected |
| **24 hours post-infection** | 2 wounds | 1 wound | 2 wounds | 1 wound |
| **48 hours post-infection** | 2 wounds | 1 wound | 2 wounds | 1 wound |
| **72 hours post-infection** | 2 wounds | 1 wound | 2 wounds | 1 wound |
| **96 hours post-infection** | 2 wounds | 1 wound | 2 wounds | 1 wound |


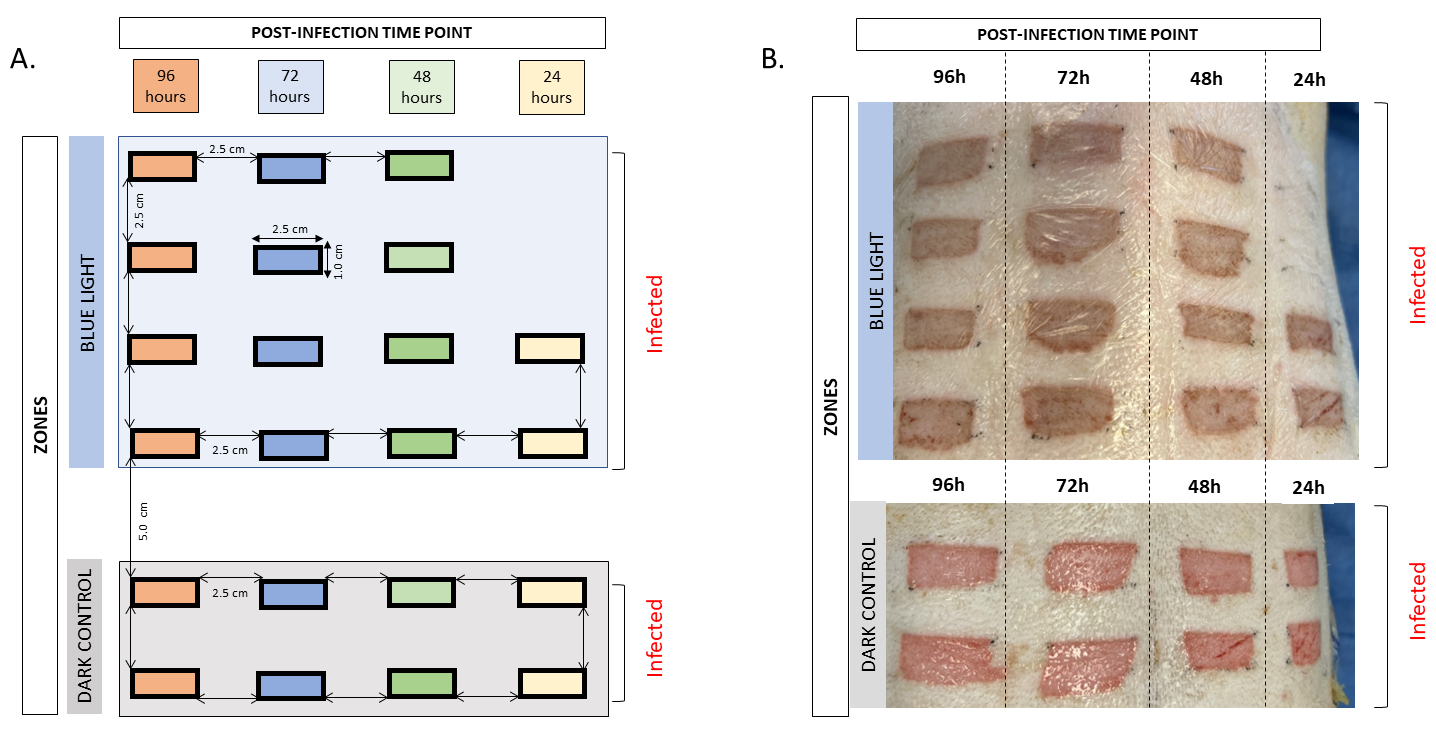


**Figure S4**. A total of 22 wounds were placed in pig #3, separated by two different “zones”: blue light zone and dark zone (control). The flowchart (A) and the dorsal chest of the pig (B) show that the wounds were divided into four different groups to grow the bacteria biofilms at different time points post-infection (24h, 48h, 72, and 96h), each group contained 4 wounds for light irradiation and 2 wounds for dark control, all infected.

**Table S2**. The table shows the number of wounds in pig #3, in different zones (blue light zone and dark control zone) for each post-infection group (24h, 48h, 72h, 96h post-infection), over a total of 22 wounds.

| ***Groups***  ***( Time post-infection)*** | ***Zones of treatment*** | |
| --- | --- | --- |
|  | **Blue light zone** | **Dark control zone** |
|  | Infected | Infected |
| **24 hours post-infection** | 4 wounds | 2 wounds |
| **48 hours post-infection** | 4 wounds | 2 wounds |
| **72 hours post-infection** | 4 wounds | 2 wounds |
| **96 hours post-infection** | 4 wounds | 2 wounds |


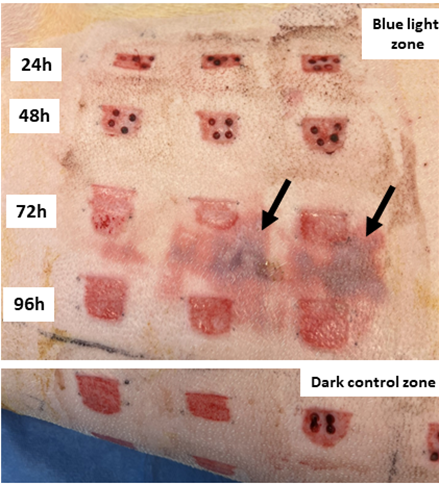


**Figure S5**. The antimicrobial blue light device (ABLD) without a cooling circuit system showed a significant increase in temperature in the first porcine model resulting in burning skin around the wounds (black arrows) after the ABL irradiation at 50 mW/cm^2^, 250 J/cm2.

**Table S3.** Log CFU/g reductions of MRSA in infected wounds for each time post-infection (24h, 48h, 72h, and 96h) in the dark control and under ABL before and after daily treatments.

|  |  | |  | | ***Log CFU/g of MRSA in infected wounds*** | | | | |
| --- | --- | --- | --- | --- | --- | --- | --- | --- | --- |
| ***Groups*** | **Dark** | ***24 hours after last aBL treatment*** | | | | | ***30 minutes after last aBL treatment*** | | |
|  |  | **Before daily treatment** | | **p values** | | **Total of ABL treatment (T)** | **After daily treatment** | **p values** | **Total of ABL treatment (T)** |
| 24h post-infection | 4.5 | - | | - | | - | 3.8 | 0.8614 | 1T |
| 48h post-infection | 6 | 4.5 | | 0.0002 | | 1T | 3.2 | 0.0044 | 2T |
| 72h post-infection | 6.9 | 3.4 | | <0.0001 | | 2T | 3.4 | <0.0001 | 3T |
| 96h post-infection | 8.5 | 4.7 | | <0.0001 | | 3T | 3.8 | <0.0001 | 4T |

**

**Figure S6.** The culture inoculum at time 0 was 2.5 x 10^6^ log CFU/g per wound. In the untreated wounds, this progressed to almost 1x10^9^ log CFU/g per wound by 96 hours post-infection.
